# Supplementary material for: Clustering of fMRI data: the elusive optimal number of clusters
Source: PeerJ. 2018 Oct 3;6:e5416. doi: 10.7717/peerj.5416 (PMC6173948; doi:10.7717/peerj.5416)
Supplement: Supplemental Information 1 [file peerj-06-5416-s001.pdf]

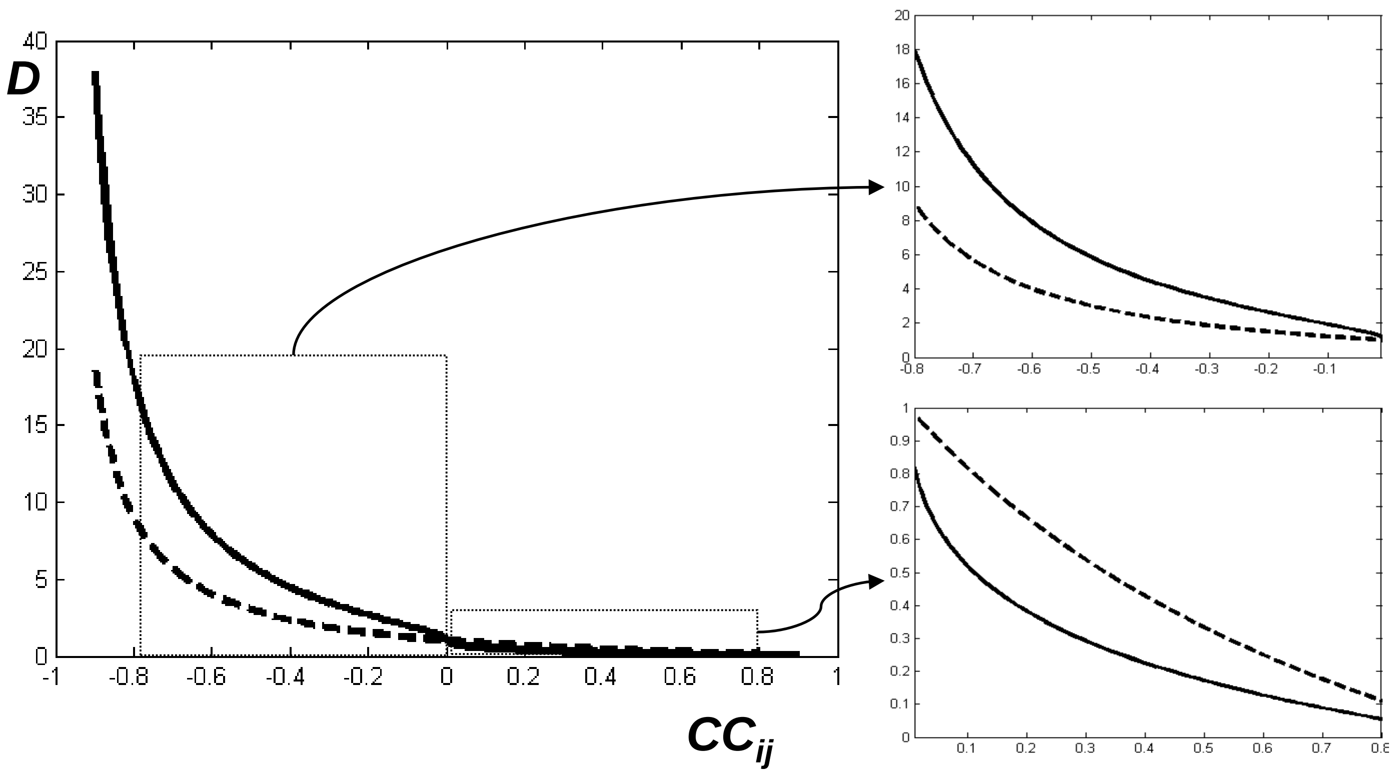

Supplementary Figure 1:

(left) similarity measure (D) as a function of the cross-correlation coefficient (CC_{ij}) using Equation (4) (dashed line) and the new formula in Equation (5) (solid line). (right) zoom in two parts of the plot (top: negative correlation; bottom: positive correlation).

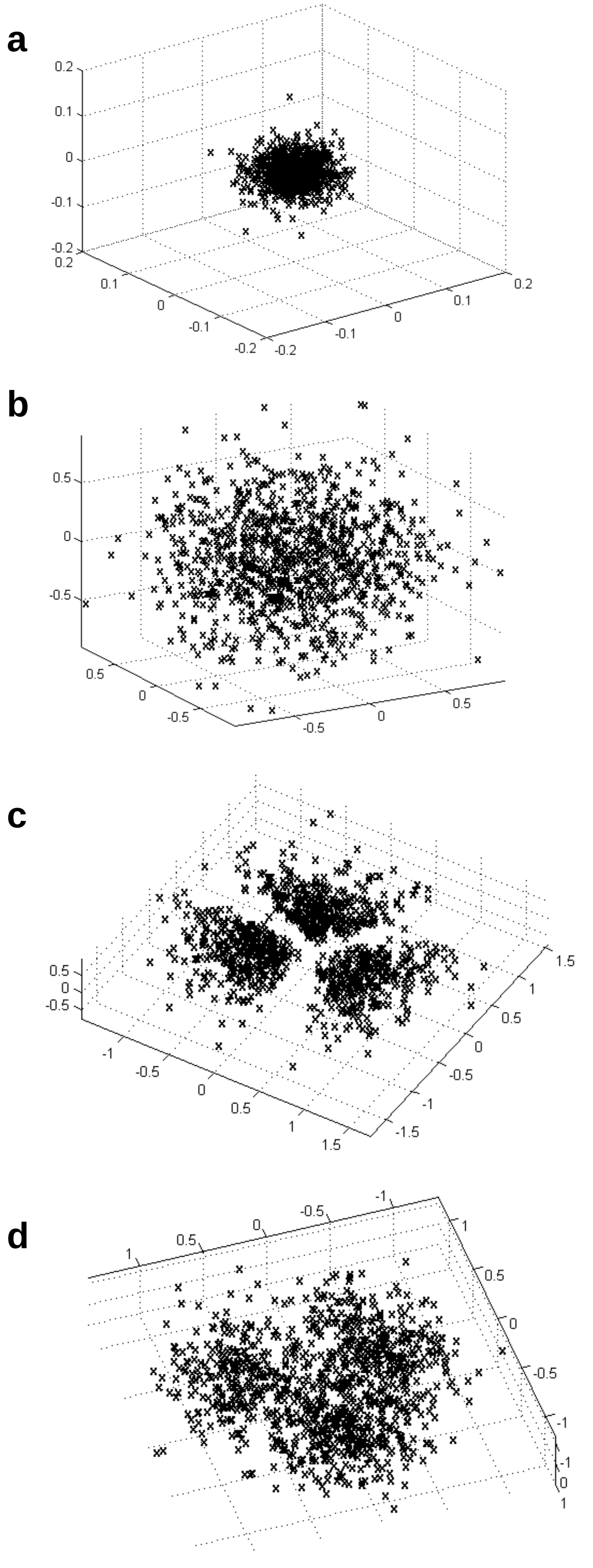

Supplementary Figure 2:

MDS 3D-plots of the simulated data: the 1-cluster dataset (a), the n-cluster dataset (b), the 3-cluster dataset with low (σ=1) noise levels (c), and the 3-clsuter dataset with high (σ=4) noise levels (D).

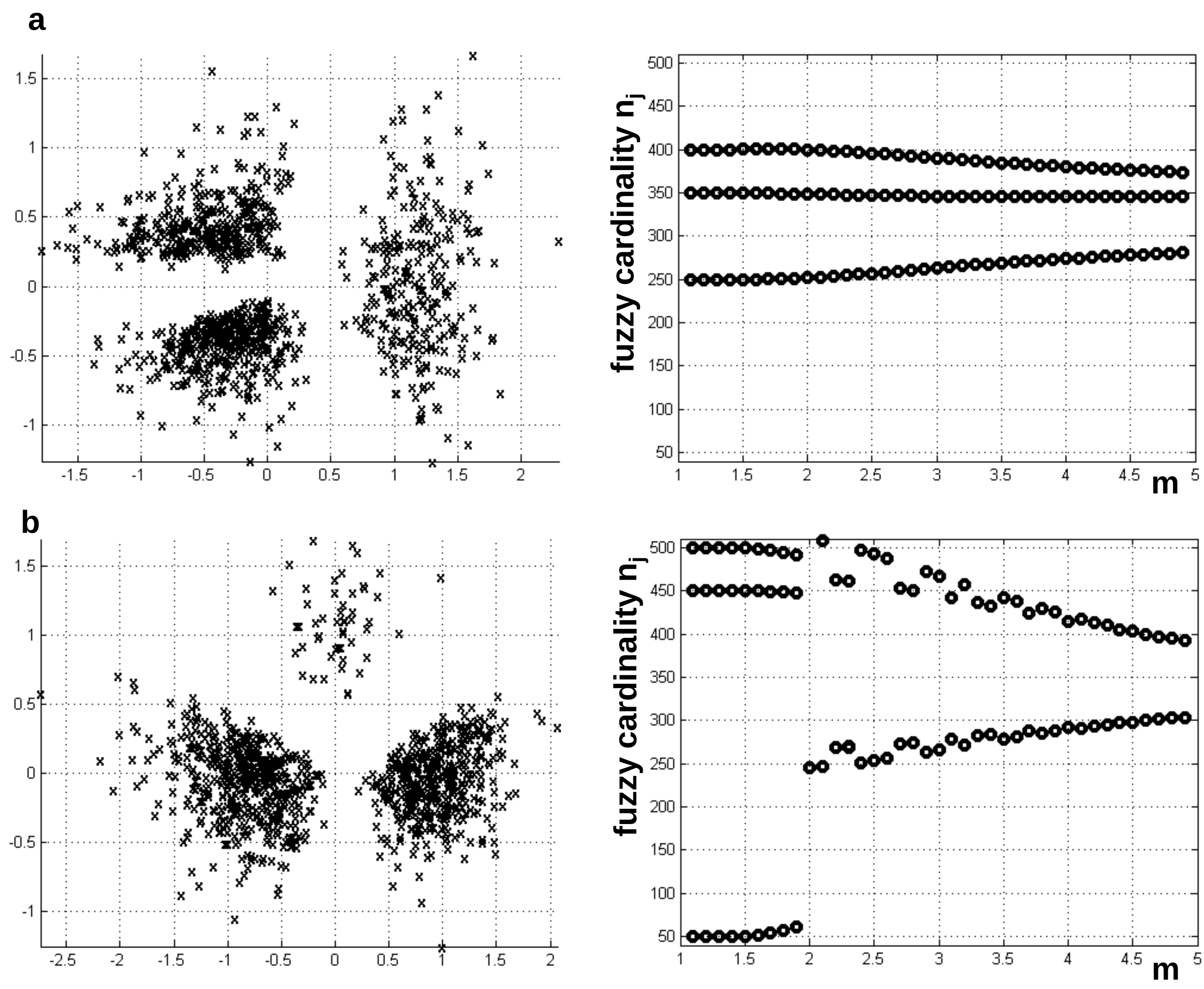

Supplementary Figure 3:
illustrates the influence of the fuzziness degree m. (a) a well-balanced dataset with 3 clusters: MDS 2D-plot of all datapoints (left) and the fuzzy cardinality of each obtained cluster at different m values (right). (b) a 3-cluster dataset with 2 dominant clusters: MDS 2D-plot of all datapoints (left) showing a cluster with small number of datapoints (less than 5% of the whole dataset), and the fuzzy cardinality of the three identified clusters at different m values (right). In (a), FCM was able to identify the three clusters at different m values, whereas in (b) the smaller cluster was not identified by FCM at higher m values (e.g. m>3).
